# Prediction of Mortality in Incident Hemodialysis Patients: A Validation and Comparison of CHADS2, CHA2DS2, and CCI Scores

**DOI:** 10.1371/journal.pone.0154627

**Published:** 2016-05-05

**Authors:** Hsun Yang, Yi-Hsin Chen, Teng-Fu Hsieh, Shiun-Yang Chuang, Ming-Ju Wu

**Affiliations:** 1 Department of Nephrology, Taichung Tzu Chi Hospital, Buddhist Tzu Chi Medical Foundation, Taichung, Taiwan; 2 Department of Urology, Taichung Tzu Chi Hospital, Buddhist Tzu Chi Medical Foundation, Taichung, Taiwan; 3 Department of Medical Research, Taichung Tzu Chi General Hospital, Buddhist Tzu Chi Medical Foundation, Taichung, Taiwan; 4 Division of Nephrology, Department of Internal Medicine, Taichung Veterans General Hospital, Taichung, Taiwan; 5 Institute of Clinical Medicine, National Yang-Ming University School of Medicine, Taipei, Taiwan; 6 School of Medicine, Tzu Chi University, Hualian, Taiwan; 7 School of Medicine, Chung Shan Medical University, Taichung, Taiwan; 8 School of Medicine, College of Medicine, China Medical University, Taichung, Taiwan; Postgraduate Medical Institute, INDIA

## Abstract

**Background:**

The CHADS2 and CHA2DS2 scores are usually applied for stroke prediction in atrial fibrillation patients, and the Charlson comorbidity index (CCI) is a commonly used scale for assessing morbidity. The role in assessing mortality with score system in hemodialysis is not clear and comparisons are lacking. We aimed at evaluating CHADS2, CHA2DS2, and CCI scores to predict mortality in incident hemodialysis patients.

**Methods:**

Using data from the Nation Health Insurance system of Taiwan (NHIRD) from 1 January 2005 to 31 December 2009, individuals ≧20 y/o who began hemodialysis identified by procedure code and receiving dialysis for > 3 months were included for our study. Renal transplantation patients after dialysis or PD patients were excluded. We calculated the CHADS2, CHA2DS2, and CCI score according to the ICD-9 code and categorized the patients into three groups in each system: 0–1, 2–3, over 4. A total of 3046 incident hemodialysis patients enrolled from NHIRD were examined for an association between the separate scoring systems (CHADS2, CHA2DS2, and CCI score) and mortality.

**Results:**

CHADS2 and CHA2DS2 scores revealed good predictive value for total mortality (CHADS2 AUC = 0.805; CHA2DS2 AUC = 0.790). However, the CCI score did not reveal a similarly satisfying result (AUC = 0.576).

**Conclusions:**

Our results show that CHADS2 and CHA2DS2 scores can be applied for mortality prediction in incident hemodialysis patients.

## Introduction

Mortality risk among hemodialysis patients may be highest soon after initiation of hemodialysis[1]. Once renal replacement therapy is initiated, the range of the expected remaining life span in the United States Renal Data System (USRDS) report is approximately 8 years for dialysis patients 40–44 years of age and approximately 4.5 years for those 60–64 years of age [2]. According to the 2011 United States Renal Data System annual data report, only 50 percent of dialysis patients, and 82 percent of those who receive a preemptive transplant, are still alive three years after the start of ESRD therapy. Identifying the period of highest risk for death after initiation of hemodialysis and also the factors that are associated with this higher risk are important to the care of incident hemodialysis patients.

Unfortunately, there is limited information available about mortality rates and factors that influence mortality immediately after hemodialysis initiation [3–6]. One population-based study found that 6% of hemodialysis patients died within the first 90 days, accounting for nearly 32% of the deaths within the first year [7]. Considering the major causes of death in the dialysis patient population that include cardiovascular disease, infection, and withdrawn from dialysis, cardiovascular disease accounts for approximately 50 percent of deaths [2].

The CHADS2 and CHA2DS2 scoring systems have been used for stroke risk stratification in atrial fibrillation patients. Previous studies have already applied this tool to predict cardiovascular mortality [8, 9], and risk assessment in recent studies performed using CHADS2 for MACEs (major adverse cardiovascular events) is feasible [10]. Further, incident uremic patients with comorbid conditions, which have been shown to deteriorate after initiating hemodialysis, were associated with increased mortality [11, 12]. Even CCI has not been adequately validated for ESRD patients, there have also been several studies that have investigated the association between the Charlson comorbidity index (CCI) score and survival on hemodialysis [13, 14]. Using the CHADS2 and CHA2DS2 scoring system may somehow avoid the limitation of CCI in ESRD patients, although these scores were not originally established for assessing ESRD patients. Therefore, the aim of this study was to explore the risk of mortality in incident hemodialysis patients by comparing different assessment scores.

Following the launch of National Health Insurance (NHI) in 1995, incidence and prevalence rates in dialysis patients in Taiwan increased rapidly [15]. To find an effective way to evaluate the mortality risk is thus an important issue for the fast-growing dialysis population. In our study, a retrospective cohort design was used to investigate the predictive value of CHAD2, CHA2DS2 and CCI scores for mortality in incident hemodialysis patients by using the National Health Insurance (NHI) claim data.

## Materials and Methods

### Data Source

The NHI program was initiated in 1995 and all medical institutions must submit standard claim documents for medical expenses on a computerized form. Admission and discharge dates, patient identification number, sex, date of birth, and International Classification of Disease, Ninth Revision, Clinical Modification (ICD-9CM) diagnostic codes for the admission and outpatient clinic visit are included. These codes consist of the principal and up to four secondary diagnoses for inpatient care and two secondary diagnoses for outpatient care. The database also includes codes for treatment procedures and materials used in healthcare. At the end of 2011, there were 23 million beneficiaries included in Taiwan’s NHI program. Because of its extensive scope that includes residential area and personal income, further comprehensive analyses of health care utilization patterns are possible. Thus, all the patients’ data of this retrospective cohort study were retrieved from the database of the Bureau of NHI, which scrambles the patients’ personal identification numbers for reason of privacy.

### Study Population

This observational study was conducted in a retrospective cohort of the Taiwanese population from the year 2005 to 2009 and who were enrolled in the NHIRD in Taiwan. The National Health Insurance Bureau of Taiwan randomly reviews the charts of 1 out of every 100 ambulatory cases and 1 out of every 20 inpatient cases. Diagnosis accuracy was also verified. Incident patients (n = 3046) ≧20 years of age who began hemodialysis (procedure codes 58001C, 58019C to 58025C) between 1 January2005 and 31 December 2009 were recruited for this study. These patients had received regular hemodialysis for over three months[16]. Renal transplantation patients after dialysis and peritoneal dialysis patients were excluded.

### Risk Score Calculation

The CHADS2 score (Table 1) were calculated for each patient by assigning 1 point each for the presence of chronic heart failure, hypertension, age≧75 years, and diabetes and by assigning 2 points of prior stroke or transient ischemic attack (TIA).

**Table 1:** CHADS2 score.

Risk Factor	Score
Chronic heart failure	1
Hypertension	1
Age≧75 years	1
Diabetes	1
Prior stroke or transient ischemic attack (TIA)	2

The CHA2DS2 score (Table 2) were calculated for each patient by assigning 1 point each for the presence of chronic heart failure, hypertension, diabetes, vascular disease, age 65–74 years, and female sex; and by assigning 2 points of age≧75 years, prior stroke or transient ischemic attack(TIA). In clinical use, the CHADS2 score has been superseded by the CHA2DS2 score that gives a better stratification of low-risk patients [17].

**Table 2:** CHA2DS2 score.

Risk Factor	Score
Chronic heart failure	1
Hypertension	1
Diabetes	1
Vascular disease	1
Age 65–74 years	1
Female sex	1
Age≧75 years	2
Prior stroke or transient ischemic attack (TIA)	2

The CCI score (CCIS, Romano–Charlson comorbidity index score, Table 3) was calculated for each patient according to 19 diagnoses obtained from a review of the patient’s medical chart. One point is assigned for each for the presence of prior myocardial infarction, congestive heart failure, peripheral vascular disease, cerebrovascular disease, dementia, chronic pulmonary disease, rheumatologic disease, peptic ulcer disease, mild liver disease or diabetes. Two points are assigned for each presence of cerebrovascular (hemiplegia) event, moderate-to-severe renal disease (zero points was set in this study because of incident hemodialysis patients), diabetes with chronic complications and cancer without metastases. Three points are assigned for moderate or severe liver disease and six points are assigned for a metastatic solid tumor or full-blown acquired immuno-deficiency syndrome (AIDS, zero points was set in this study because our dataset contained no AIDS patients regularly using hemodialysis).

**Table 3:** CCI (Charlson comorbidity index) score.

Comorbidity	Score
Prior myocardial infarction	1
Congestive heart failure	1
Peripheral vascular disease	1
Cerebrovascular disease	1
Dementia	1
Chronic pulmonary disease	1
Rheumatologic disease	1
Peptic ulcer disease	1
Mild liver disease	1
Diabetes	1
Cerebrovascular (hemiplegia) event	2
Moderate-to-severe renal disease	2
Diabetes with chronic complications	2
Cancer without metastases	2
Leukemia	2
Lymphoma	2
Moderate or severe liver disease	3
Metastatic solid tumor	6
Acquired immuno-deficiency syndrome (AIDS)	6

The scores for individual conditions were summed to yield a total score for predicting mortality. The study patients were divided into three groups by their score points: group 1(0–1 point), group 2(2–3 points), group 3(over 4 points) in each CHADS2, CHA2DS2, and CCI score system.

### Study End Point and Patient Follow-up

The survival time was calculated 90 days after starting hemodialysis. Mortality data covering the years from 2005 to 2009 were used to calculate the mortality rate in each group. Each patient was tracked for five years from the time of starting hemodialysis.

### Statistical Analysis

SAS for Windows (version 9.2, SAS Institute Inc., Cary, NC, USA) was used to perform data analysis and SPSS (version 19.0, SPSS Inc., Chicago, IL, USA) was used to plot the survival curves. A receiver operating characteristics curve was used to assess the prediction accuracy for mortality by using the CHADS2, CHA2DS2, and CCI scores. Plots of observed and predicted mortality were formulated. The cumulative rates of mortality were estimated using the log rank test to examine the differences in the risk of mortality between different groups among the incident hemodialysis patients. The Cox proportional hazards regression model was used to compare the outcomes between different risk groups. We calculated hazard ratios (HR) along with 95% confidence intervals (CI) using a significance level of 0.05. A two- sided p value (p<0.05) was used to determine statistical significance. Furthermore, each comorbidity in the CHADS2 and CHA2DS2 score was identified as separate variable and further analyzed in this study.

## Results

There were 3046 defined incident hemodialysis patients enrolled in the NHIRD of Taiwan during year 2005 to 2009. We divided the patients into three groups by comorbidity severity based on scores (group 1: 0–1, group 2: 2–3, group 3: over 4). The numbers of patients, age, gender and distribution of number in different CHADS2, CHA2DS2 and CCI score groups are shown in Table 4. The mean age was 61±13 years and 51% of patients were female. The median of the CHADS2 score was 1 (range 0–6), the median of CHA2DS2 score was 2 (range 0–9), and the median of CCI score was 0 (range 0–11). Severe comorbidity was noted in the different scoring systems as group 3 in 7.6%, 21.5%, and 4.5% of all enrolled patients, respectively.

**Table 4:** Baseline characteristics of the incident hemodialysis patients from 2005 to 2009 in Taiwan.

Variables	Study population, N (%)
Total	3046
Mean age,years(±SD)	61±13
CHADS2 score	
Median(range)	1(0–6)
Group 1: 0–1	2021(66.3)
Group 2: 2–3	795(26.1)
Group 3: Over 4	230(7.6)
CHA2DS2 score	
Median(range)	2(0–9)
Group 1: 0–1	1155(37.9)
Group 2: 2–3	1237(40.6)
Group 3: Over 4	654(21.5)
CCI score	
Median(range)	0(0–11)
Group 1: 0–1	2286(75.0)
Group 2: 2–3	625(20.5)
Group 3: Over 4	135(4.5)
Gender	
Male	1492(49.0)
Female	1554(51.0)
Comorbidities	
Hyperlipidemia	322(10.6)
Coronary artery disease	471(15.5)
Atrial fibrillation	48(1.6)
Socioeconomic status	
Disadvantaged SES	2593(85.1)
Advantaged SES	453(14.9)
Urbanization	
Urban	843(27.7)
Nonurban	2203(72.3)
Geographic region	
North-West	1869(61.4)
South-East	1177(38.6)

**Fig 1:**
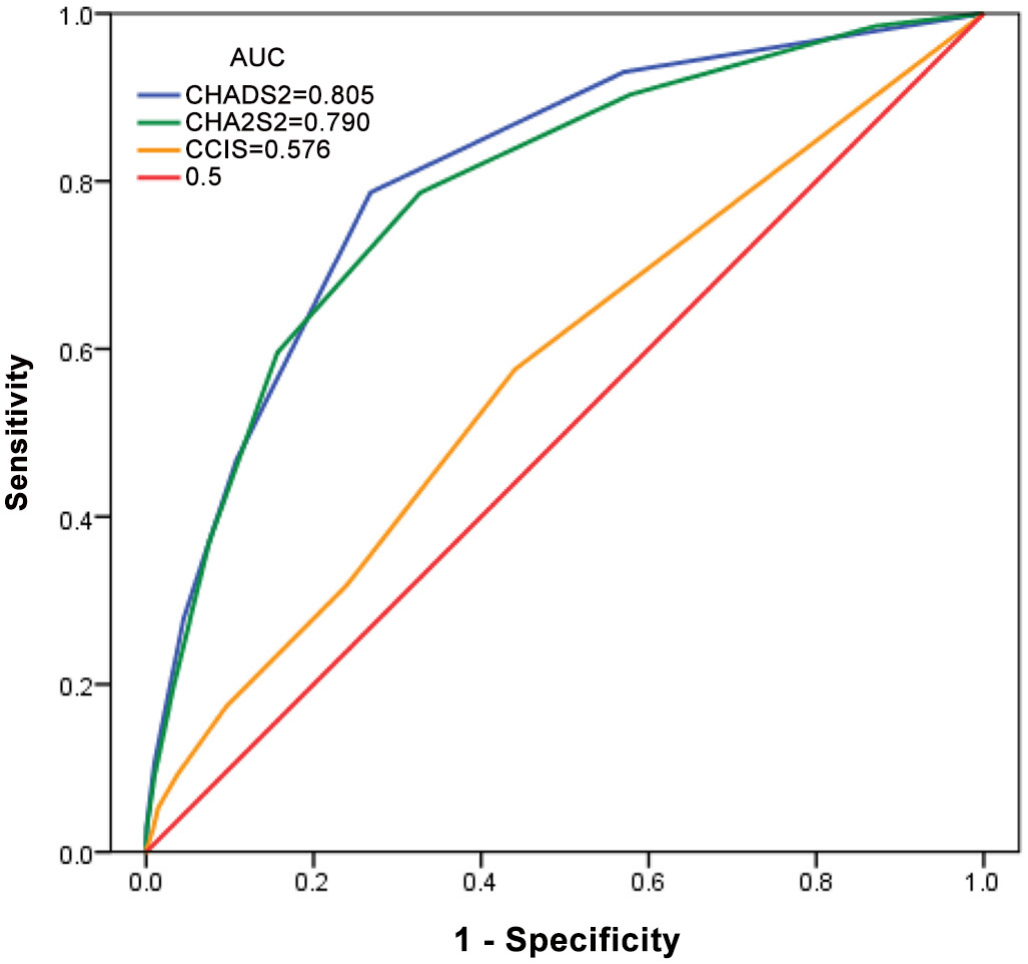
Receiver operating characteristics curve for CHADS2, CHA2DS2 and CCI in the prediction of mortality in incident hemodialysis patients.

Table 5 presents our further analysis of the cumulative rate of mortality for five years in the different CHADS2, CHA2DS2, and CCI scores. Incident hemodialysis patients with higher CHADS2, CHA2DS2, and CCI scores had a higher mortality rate. Fig 1 shows that the area under curve (AUC) was 0.805 in the CHADS2 stratified groups (95% CI, 0.783–0.828), 0.790 in the CHA2DS2 stratified groups (95% CI, 0.766–0.814), and 0.576 in the CCI stratified groups (95% CI, 0.545–0.606).

**Table 5:** The cumulative rate of mortality among different CHADS2, CHA2DS2 and CCI scores over five years.

Variables	n	Case(%)	p-value
CHADS2 score			<0.001
Group 1: 0–1	2021	86(4.3)	
Group 2: 2–3	795	205(25.8)	
Group 3: Over 4	230	112(48.7)	
CHA2DS2 score			<0.001
Group 1: 0–1	1155	39(3.4)	
Group 2: 2–3	1237	124(10.0)	
Group 3: Over 4	654	240(36.7)	
CCI score			<0.001
Group 1: 0–1	2286	275(12.0)	
Group 2: 2–3	625	91(14.6)	
Group3: Over 4	135	37(27.4)	

In the multivariate analysis, each additional CHADS2 score was associated with a 1.90-fold (95% CI, 1.79–2.02) increased mortality rate, each additional CHA2DS2 score was associated with a 1.76-fold (95% CI, 1.66–1.86) increased mortality rate, and each additional CCI score was associated with a 1.22-fold (95% CI, 1.15–1.30) increase when CHADS2, CHA2DS2, and CCI scores were a continuous variables (Table 6, model A). In model B of Table 6, where the CHADS2 score was an ordinal variable, patients with a higher score remained as an independent prognostic factor for the risk of mortality with hazard ratios of 6.69 (95% CI: 5.19–8.64, P<0.001) in group 2 and 14.03 (95% CI: 10.51–18.74, P<0.001) in group 3, compared with the CHADS2 score group 1 after adjusting for other factors. While the CHA2DS2 score was an ordinal variable, the hazard ratios of 3.44 (95% CI: 2.35–5.02, P<0.001) in group 2 and 15.64 (95% CI: 10.78–22.71, P<0.001) in group 3 were also noticed, compared with the CHA2DS2 score of group 1 after adjustments. And in the CCI score groups, while the CCI score was an ordinal variable, only in group 3 was it statistically significant with hazard ratios of 2.76 (95% CI: 1.95–3.91, P<0.001) compared with the CCIS score of group 1 after adjusting for other clinical covariates.

**Table 6:** Hazard ratios of individual CHADS2, CHA2DS2 and CCI scores for mortality in incident hemodialysis patients.

	Model A^a^			Model B^b^		
	Adjusted HR	95% CI	*P* value	Adjusted HR	95% CI	p-value
CHADS2 score	1.90	1.79–2.02	<0.001			
Group 1: 0–1	1			1		
Group 2: 2–3				6.69	5.19–8.64	<0.001
Group 3: Over 4				14.03	10.51–18.74	<0.001
CHA2DS2 score	1.76	1.66–1.86	<0.001			
Group 1: 0–1	1			1		
Group 2: 2–3				3.44	2.35–5.02	<0.001
Group 3: Over 4				15.64	10.78–22.71	<0.001
CCI score	1.22	1.15–1.30	<0.001			
Group 1: 0–1	1			1		
Group 2: 2–3				1.26	0.99–1.61	0.059
Group 3: Over 4				2.76	1.95–3.91	<0.001

Adjusted for the patients' age, gender, hyperlipidemia, coronary artery disease, atrial fibrillation, socioeconomic status, urbanization and geographic region.

^a^score as continuous variable.

^b^score as ordinal variable.

**Fig 2:**
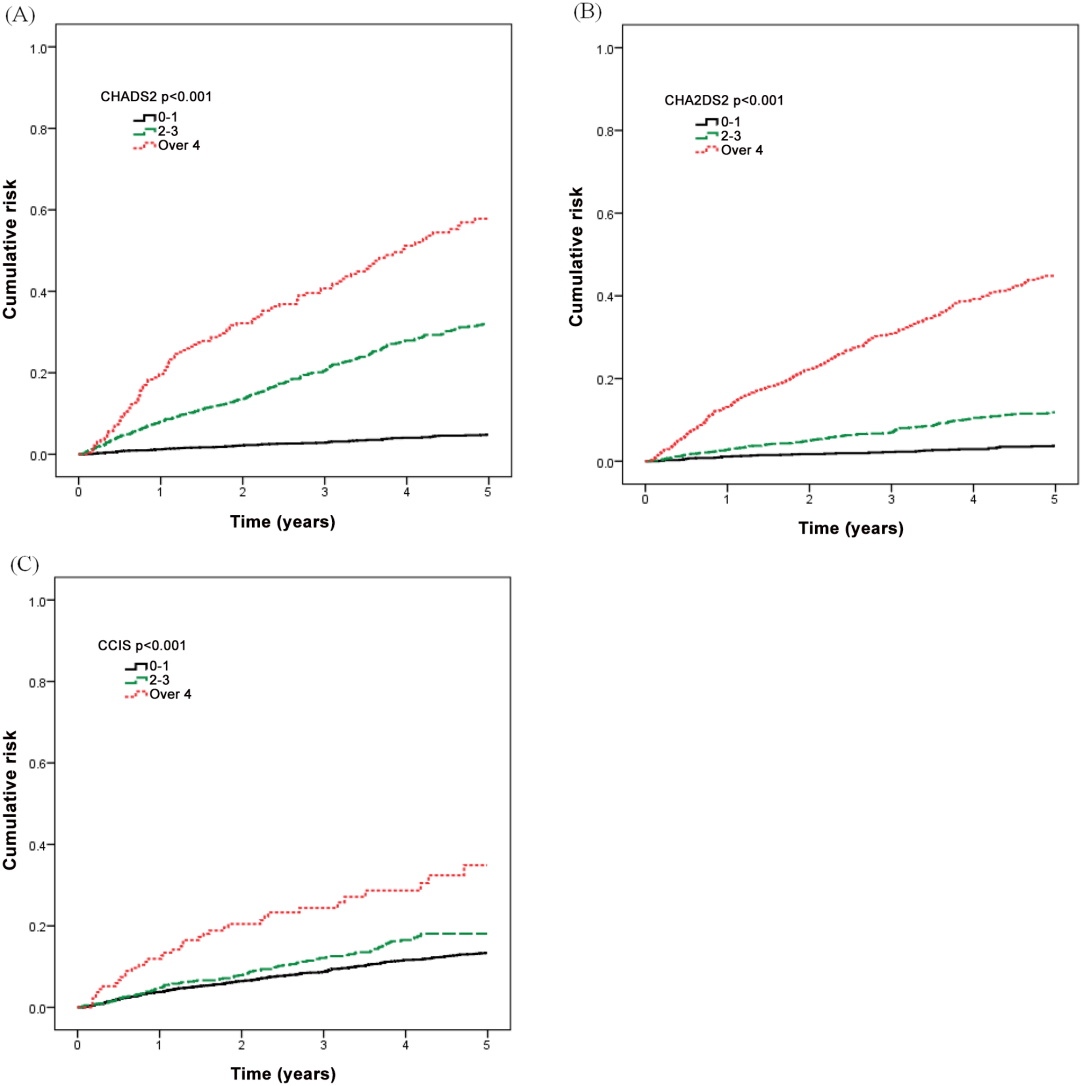
Mortality risk stratified by CHADS2, CHA2DS2, and CCI score categories. (A) CHADS2 score. (B) CHA2DS2 score. (C) CCI score.

The mortality risk stratified by CHADS2, CHA2DS2, and CCI score categories was compared using a Kaplan-Meier survival analysis (Fig 2). Incident hemodialysis patients with higher CHADS2, CHA2DS2, and CCI scores had a higher mortality rate (log rank test P<0.001). When further analyzing each comorbidity, hypertension was associated with the greatest increased risk for mortality in the CHADS2 score (Table 7, adjusted HR 2.98, 95% CI 2.29–3.87, p<0.001), and in the CHA2DS2 score (Table 8, adjusted HR 2.87, 95% CI 2.20–3.74, p<0.001).

**Table 7:** Hazard ratios of individual comorbidity of the CHADS2 score for mortality in incident hemodialysis patients.

	Adjusted HR	95% CI	*P* value
Congestive heart failure	2.18	1.74–2.72	<0.001
Hypertension	2.98	2.29–3.87	<0.001
Diabetes mellitus	2.12	1.71–2.64	<0.001
**S**troke/transient ischemic attack/thromboembolism	2.33	1.87–2.91	<0.001

**Table 8:** Hazard ratios of individual comorbidity of the CHA2DS2 score for mortality in incident hemodialysis patients.

	Adjusted HR	95% CI	*P* value
Congestive heart failure	2.02	1.60–2.56	<0.001
Hypertension	2.87	2.20–3.74	<0.001
Diabetes mellitus	2.07	1.67–2.58	<0.001
**S**troke/transient ischemic attack/thromboembolism	2.30	1.84–2.87	<0.001
Vascular disease	1.29	1.01–1.63	0.039

## Discussion

The present study is the first study designed to compare the mortality risk by assessment of CHADS2, CHA2DS2, CCI scores in incident hemodialysis patients. The main finding of this study is that the CHADS2 and CHA2DS2 scores are effective in predicting mortality. Although the CCI score had an unsatisfactory AUC, there was still significant difference between groups in the cumulative rate of mortality for five years (Table 5). Moreover, the higher CCI score group (group 3) also had a significant HR (2.76, CI: 1.95–3.91) in the Cox proportional hazards regression model (Table 6, model B). We validated the application of the CHADS2 and CHA2DS2 scores for mortality of the incident dialysis patients to check the accuracy of our prediction, which was 80.5% and 79.0%, respectively. Hypertension, which presented in approximately 80% of patients at the onset of dialysis and approximately 25 to 30% at the end of the first year, was associated with the greatest increased risk for mortality in the following analyses (Tables 7 & 8) as compared to each comorbidity disease of the CHADS2 & CHA2DS2 scores.

In clinical practice, the CHADS2 score is useful in guiding antithrombotic therapy for atrial fibrillation, with patients having a high score benefiting from anticoagulation therapy. The CHA2DS2 score has been proved to be more sensitive than the CHADS2 score in discriminating between patients at low risk of cardio-embolic events [18], and it is indicated in the recent ESC Guidelines [19] as the preferred instrument to guide the choice of anticoagulant treatment. However, there is little direct evidence of the benefit of antithrombotic agents in the prevention of stroke, cardiovascular events, or vascular access thrombosis in dialysis patients [20–26]. Hemodialysis (HD) patients are at a higher risk of serious bleeding due to several factors including uremic platelet dysfunction, anemia, and heparin use during dialysis[27–30]. Therefore, in dialysis patients clinical assessment scoring system such as CHADS2 & CHA2DS2 may help physicians to identify patients at a higher risk for unfavorable events, and establish preemptive monitoring programs in order to decrease disease severity and negative outcomes instead of guiding therapy.

Comorbidity is an important predictor for mortality. However, after adjusting for age, gender, primary renal disease, treatment modality and geographic region, the influence of comorbidity in survival between patient groups may be less important than is generally expected [31]. This may explain why the CCI score in our study has relatively less predictive value.

The CCI was initially developed in a cohort of 559 medical patients and was tested for its ability to predict risk of death from comorbid disease in the second cohort of 685 patients during a 10-yr follow-up which published at 1987.[32] However, the CCI does have limitations when applied to ESRD patients for renal disease is one of the comorbidity diseases. Thus, to avoid some limitation of original CCI, the End Stage Renal Disease Comorbidity Index (ESRD-CI) was created [33], which included 11 cormobidities instead of 19 cormorbidities in CCI. But, in the end the model of ESRD-CI had only slightly better performance compared to the CCI (c-statistic of 0.73 versus 0.72). Recently, one contemporary Canadian cohort tried to assess the validity of these two comorbid indicies (CCI & ESRD-CI) using more recent era clinical data (2006–2013) [34]. It was discussed that the reduced prevalence of overall cormorbidities and the relative impact of some comorbid conditions may have changed, such as AIDS, cancer, myocardial infarction, DM type 1, etc,[35–38] those improvements may affect the predict value of CCI & ESRD-CI (c-statistic of 0.61 versus 0.63) compared to the derivation cohorts. Therefore, a further validation of more useful scores other than previously derived comorbidity indices in contemporary dialysis cohorts may be needed to exam. Considering the variation of 19 comobidities of CCI and 11 comobidities of ESRD-CI, the variable factor was 5 items in CHADS2 and 8 items in CHA2DS2, which were easily to obtain and cause less bias. We hoped that our study could re-exam and introduced an intuitive using of existent score system to mortality assessment in ESRD patients by using a simple, work well, easy to use and widely used score system with better performance.

There have been many reports on mortality investigations and analyses in incident hemodialysis patients [39–42]. However, a focus on score evaluation remains scarce. The strength of our data is based on the fact that it was a nationwide population-based study and the dataset was routinely monitored for diagnostic accuracy by the National Health Insurance Bureau of Taiwan. The National Health Insurance (NHI) program requires mandatory enrollment in the government-run, universal, single-payer insurance system and provides comprehensive nationwide coverage. Taiwan’s NHI research database is one of the largest and most reliable databases of its kind, and has been widely used in many previous studies.

Our study nonetheless has several limitations related to the NHIRD. First, there may be inaccuracies in the recording of the diagnostic and therapeutic codes, but a previous study of a Taiwanese medical center indicated about 95% consistency between patient records and claims data, which offers evidence of the validity and acceptable accuracy of the NHIRD [43]. Second, we could not obtain body height, weight, education, occupation, information on personal habits, disease severity, or family history because of subjects’ privacy is protected by the NHI research database. Third, the laboratory data could not be obtained, so we could not know the dialysis adequacy and basic biochemical data. This scoring system would be of much value if clinical data can be included and analyzed for risk stratification rather than using simple diagnosis category for assessing mortality risk. Therefore this scoring system only added limited clinical assessment value because those factors are already known risks for hemodialysis patients. Fourth, the CHADS2 & CHA2DS2 scores were not originally established for assessing ESRD patients, and these scores may not have adequate discriminatory value between ESRD patients. However, the c-statistic of CCI score was low and has not been adequately validated in ESRD group; we may use the CHADS2 & CHA2DS2 scores in ESRD patients instead of its original design for antithrombotic therapy. Fifth, we could not compare the causes of death, which were also not available from the NHIRD. Finally, the design of this study is retrospective, and the limitation of scores’ validation in retrospective cohort studies always weakens the strength of the results.

However, we believe that the relatively large number of patients may compensate for above-mentioned limitations. According to the United States Renal Data System, the prevalence of ESRD reached 2,584 per million in 2010 in Taiwan, while rates of 2,260 and 1,870 were reported in Japan and the U.S. Incident rates of reported ESRD in 2010 were 3361, 369 and 288 per million population in the Taiwan, United States, and Japan, respectively. Our study population was close to the incident rate of Taiwan and reflects the real situation.

## Conclusion

CHADS2 and CHA2DS2 scores could be a useful tool to predict the mortality in incident hemodialysis patients in our study. Hypertension was the most important factor for mortality prediction in the CHADS2 and CHAD2DS2 scores. For high score (>4) groups, further mortality risk evaluation and management should be applied more aggressively.
